# The association between systemic inflammatory response index and in‐hospital mortality in patients with infective endocarditis

**DOI:** 10.1002/clc.23829

**Published:** 2022-04-11

**Authors:** Zhenzhen Cai, Tengfei Qiao, Ying Chen, Mengxiao Xie, Jun Zhou

**Affiliations:** ^1^ Department of Laboratory Medicine The First Affiliated Hospital of Nanjing Medical University Nanjing Jiangsu China; ^2^ Branch of National Clinical Research Center for Laboratory Medicine Nanjing Jiangsu China; ^3^ Department of Laboratory Medicine Nanjing Lishui District Hospital of traditional Chinese medicine Nanjing Jiangsu China

**Keywords:** infective endocarditis, prognosis, SIRI

## Abstract

**Background:**

Infective endocarditis (IE) has a significant mortality, and early identification of high‐risk patients and prediction of poor outcomes is of great significance. In recent years, increasing research has revealed the predictors associated with infective endocarditis prognosis. Systemic inflammatory response index (SIRI) is an important new indicator of inflammation. So far, there have been no reports on the relationship between SIRI and the prognosis of IE patients.

**Hypothesis:**

The purpose of this study was to explore the value of SIRI in predicting in‐hospital death for patients with infective endocarditis (IE), so as to provide reference for improving the prognosis of patients with IE.

**Method:**

A retrospective analysis was performed on the clinical data of patients with IE admitted to the First Affiliated Hospital of Nanjing Medical University from January 2017 to December 2019. SIRI was calculated according to the blood routine results of patients at admission; receiver operating characteristic curve was employed to determined the optimal cutoff value of SIRI. Patients were divided into groups (low SIRI group and high SIRI group; nonsurvivor group and survivor group) according to the levels of SIRI or their prognosis, and the general clinical features of the two groups were compared. Univariate and multivariate logistic regression analysis were performed to analyze the independent prognostic factors of in‐hospital death in IE patients.

**Results:**

A total of 147 IE patients meeting the diagnostic criteria were included, including 102 males (69.4%) and 45 females (30.6%). There was statistically significant difference in SIRI level between nonsurvivor group and survivor group (p < .05). After adjusting for the related factors, the risk of in‐hospital death in the high SIRI was still a risk of in‐hospital death with statistical significance (hazard ratio = 5.053, 95% confidence interval: 1.426‒17.905, p = .012).

**Conclusions:**

Higher SIRI level is independently associated with the risk of in‐hospital death in IE patients, and can be an independent predictor of poor outcome in IE patients.

## 1 INTRODUCTION

Infective endocarditis (IE) is an inflammation of the heart valve or lining of the ventricle wall caused by infection of the endocardial structure by bacteria, fungi or other microorganisms. IE is a fatal systemic disease that occurs in 3−10 persons per 100 000.^1^

In recent years, with the rapid improvement of the medical level, the treatment of IE has made great progress, but there are still high mortality and various serious complications. Among infectious diseases, IE ranks the top three in terms of mortality, with a 6‐month mortality rate of 20%,^2^ and more than one‐third of patients die within 1 year.^1^ Rapid identification of patients at high risk of death can provide opportunities to change the development process of the disease and improve prognosis. Therefore, early diagnosis and interventional treatment of IE are particularly important.

A number of potential biomarkers have been proposed to reflect the complex pathophysiological mechanisms of disease processes, including proinflammatory and anti‐inflammatory processes, humoral and cellular immune responses. The severity of inflammation can be indicated by the degree of increase or decrease in markers such as white blood cell count (WBC), C‐reactive protein (CRP), procalcitonin, erythrocyte sedimentation rate.^3^ In recent years, more and more new inflammatory markers, including peripheral blood cell parameters and their ratios, have been discovered and became the focus of many researchers and clinicians. In 2016, systemic inflammatory response index (SIRI) was first proposed to be employed as an independent factor influencing the prognosis of patients with advanced pancreatic cancer.^4^ Compared with a single inflammatory indicator, these composite indicators are more stable and less susceptible than other factors. To date, there have been no studies on the relation between SIRI and outcomes in IE patients. Thus, we explored the value of SIRI in predicting in‐hospital death for patients with IE, so as to provide reference for improving the prognosis of patients with IE.

## 2 DATA AND METHODS

### 2.1 Study population

The clinical data of patients with IE treated in the First Affiliated Hospital of Nanjing Medical University from January 2017 to December 2019 were collected. A total of 147 patients meeting the improved Duke diagnostic criteria^5^ were included in the study. All patients (≥18 years old) had complete laboratory data, no history of malignant tumors, no blood system, and immune system diseases. This study was approved by the Ethics Committee of the First Affiliated Hospital of Nanjing Medical University.

### 2.2 Methods

Clinical data including general data, laboratory and microbial tests, treatment methods, efficacy, and outcome were collected. The laboratory test results were taken from the venous blood test of the patient at the first admission and blood count was determined using an automatic blood cell counter (XN‐9000 assembly line; Sysmex). Biochemical results were analyzed using an automatic biochemical analyzer (AU5800 Automatic Biochemical analyzer, Beckman Coulter). LWR, NLR, PLR, SII and SIRI were calculated using the methods in the literature (LWR = Lym/WBC, NLR = Neu/Lym, PLR = PLT/Lym, SII = PLT × Neu/Lym, SIRI = Neu × Mo/Lym, Neu: neutrophil, Lym: lymphocyte, Mo: monocyte, PLT: platelet).^6^, ^7^

### 2.3 Follow‐up

All cause death within 30 days was the main outcome.

### 2.4 Statistical method

SPSS 21.0 statistical software was used for data analysis. *T‐*test was used for continuous variables (normal distribution), and Mann−Whitney *U* test was used for continuous variables (non‐normal distribution). Receiver operating characteristic curve (ROC) was employed to explore the performance of SIRI. Logistic regression was used to analyze the independent risk factors of in‐hospital death in IE patients, and to explore the correlation between SIRI level and the prognosis of IE patients. The results were expressed by hazard ratio (HR) with a confidence interval (CI) of 95%, and *p* < .05 was considered statistically significant.

## 3 RESULT

### 3.1 The optimal cutoff value of SIRI

**Figure 1:**
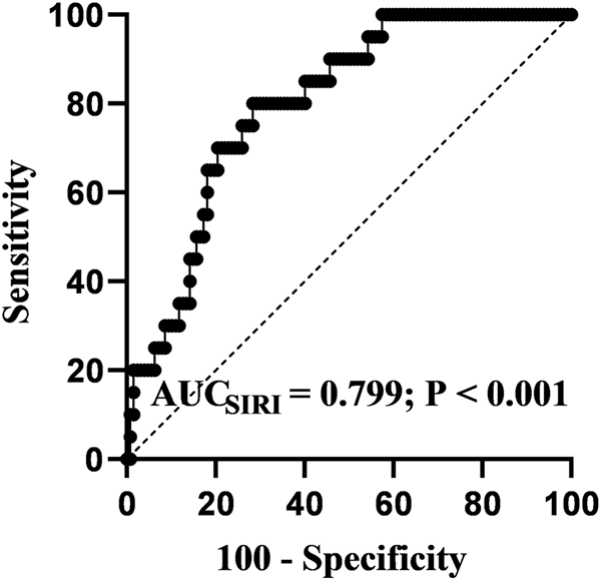
Receiver operating characteristic curve analysis of systemic inflammatory response index (SIRI)

A total of 147 IE patients were enrolled in this study, ROC was employed to determine the optimal cutoff value of SIRI, the result showed that the AUC of SIRI was 0.799 (0.725−0.861), *p* < .001; when the optimal level of SIRI (3.75) was selected, the sensitivity and specificity were 80.00% and 71.65%, respectively (Figure 1).

### 3.2 Clinical characteristics of IE patients with different SIRI levels

According to the optimal level of SIRI, patients were divided into low SIRI group and high SIRI group. The clinical characteristics of the two groups were compared, There were no statistically significant differences in gender, hemoglobin (HB), PLT, alanine aminotransferase (ALT), aspartate aminotransferase (AST), and PLR levels between the two groups. The age, WBC, Mo, Neu, urea nitrogen (UREA), creatinine (CREA), NLR, SII, and mortality of patients in the high SIRI group were significantly higher than those in the low SIRI group, and the differences were statistically significant. Lym, LWR, and surgery of patients with high SIRI were significantly lower than those with low SIRI, and the differences were statistically significant (Table 1).

**Table 1:** Clinical characteristics of study population

Variables	Low‐ SIRI	High‐ SIRI	*p* Value
	(*N* = 95)	(*N* = 52)	
Median age (years)	48.92 ± 14.62	54.71 ± 13.44	.019
Male (*N*, %)	62 (65.3%)	40 (76.9%)	.144
WBC (×10^9^/L)	7.73 ± 2.52	13.42 ± 5.99	<.001
Lym (×10^9^/L)	1.47 ± 0.70	1.08 ± 0.50	<.001
Mo (×10^9^/L)	0.45 ± 0.19	0.80 ± 0.45	<.001
Neu (×10^9^/L)	5.67 ± 2.04	11.43 ± 5.66	<.001
HB (g/L)	106.94 ± 20.63	104.54 ± 18.63	.487
PLT (×10^9^/L)	210.18 ± 119.96	176.85 ± 88.10	.081
ALT (U/L)	22.7 (6.1−280.20)	24.8 (3.00−2433.30)	.556
AST (U/L)	23.3 (10.3−218.60)	27.5 (9.10−3654.20)	.146
UREA (mmol/L)	6.01 ± 4.14	8.64 ± 5.56	.001
CREA (mmol/L)	67.3 (37.10−944.20)	81.7 (47.10−902.00)	<.001
NLR	3.95 (1.01−17.15)	9.33 (4.50−72.53)	<.001
PLR	162.32 ± 88.42	195.34 ± 141.86	.087
LWR	19.44 ± 7.90	8.83 ± 3.84	<.001
SII	749.02 (141.79−4398.06)	1614.67 (312.88−10009.6)	<.001
Mortality (%)	4.21	30.8	<.001
Surgery (%)	84.2	59.6	.001

Abbrebiations: ALT, alanine aminotransferase; AST, aspartate aminotransferase; CREA, creatinine; HB, hemoglobin; Lym, lymphocyte; LWR = Lym/WBC; Mo, monocyte; Neu, neutrophil; NLR = Neu/Lym; PL, platelet; PLR = PLT/Lym; SII = PLT × Neu/Lym; UREA, urea nitrogen; WBC, white blood cell.

### 3.3 Clinical characteristics of IE patients in the survivor group and nonsurvivor group

Comparison of general clinical characteristics of patients in the two groups (survivor group and nonsurvivor group), the results showed that there were statistically significant differences in age, WBC, Neu, UREA, CREA, LWR, NLR, SII, SIRI, and whether surgery (Table 2).

**Table 2:** Clinical characteristics of study population

Variable	Nonsurvivor group	Survivor group	*p* Value
	(*N* = 20)	(*N* = 127)	
Age (years)	57 ± 11.74	50 ± 14.63	.044
Gender (male, *n *%)	16 (80.0%)	86 (67.7%)	.271
WBC (×10^9^/L)	12.68 ± 5.79	9.28 ± 4.61	.004
Lym (×10^9^/L)	1.12 ± 0.93	1.36 ± 0.61	.131
Mo (×10^9^/L)	0.70 ± 0.34	0.55 ± 0.35	.084
Neu (×10^9^/L)	10.60 ± 5.66	7.25 ± 4.31	.002
HB (g/L)	102.70 ± 13.75	106.62 ± 20.71	.415
PLT (×10^9^/L)	175.40 ± 105.34	202.01 ± 111.37	.319
ALT (U/L)	22.7 (3.9−2433.3)	24.1 (3.0−280.2)	.468
AST (U/L)	27.5 (13.7−3654.2)	24.2 (9.1−218.6)	.360
UREA (mmol/L)	10.00 ± 6.23	6.45 ± 4.43	.002
CREA (mmol/L)	83.8 (50.7−902.0)	69.7 (37.1−944.2)	.028
LWR	0.10 ± 0.06	0.17 ± 0.08	.001
PLR	200.61 ± 132.95	166.85 ± 106.52	.205
NLR	9.33 (2.01−64.52)	5.02 (0.05−72.53)	<.001
SII	1133.27 (374.47−9613.26)	907.76 (141.79−10009.60)	.029
SIRI	5.21 (1.97−53.33)	2.39 (0.04−56.34)	<.001
Culture positive, *n* (%)	8 (40.00%)	43 (33.86%)	.593
Culture negative, *n* (%)	12 (60.00%)	84 (66.14%)	.595
Surgery (%)	40.00%	81.11%	<.001

Abbreviations: ALT, alanine aminotransferase; AST, aspartate aminotransferase; CREA, creatinine; HB, hemoglobin; Lym, lymphocyte; LWR = Lym/WBC; Mo, monocyte; Neu, neutrophil; NLR = Neu/Lym; PLT, platelet; PLR = PLT/Lym; SII = PLT × Neu/Lym; SIRI = Neu × Mo/Lym UREA, urea nitrogen; WBC, white blood cell.

### 3.4 Logistic regression analysis of in‐hospital death risk in IE patients

The factors in Table 2 with *p* < .1 were transformed into categorical variables based on optimal cutoff value. Logistic regression analysis was used to analyze the prognostic factors of IE patients. Univariate analysis showed that age, surgery, WBC, Neu, LWR, UREA, CREA, NLR, SII, and SIRI were associated with an increased risk of death in IE patients (Table 3). When the above factors were included in multivariate analysis, the results showed that, after adjusting for age, gender, or age, gender, WBC, Neu, LWR, UREA, CREA, NLR, and SII, variable SIRI was still significant in predicting the risk of in‐hospital death HR = 5.053, 95% CI: 1.426−17.905, *p* = .012) (Table 4).

**Table 3:** Univariate analyses of factors which affect the in‐hospital mortality of IE

Variables	Univariate analysis			Forest plot
	HR	95% CI	*p* Value	
Age	10.427	1.351−80.460	.004	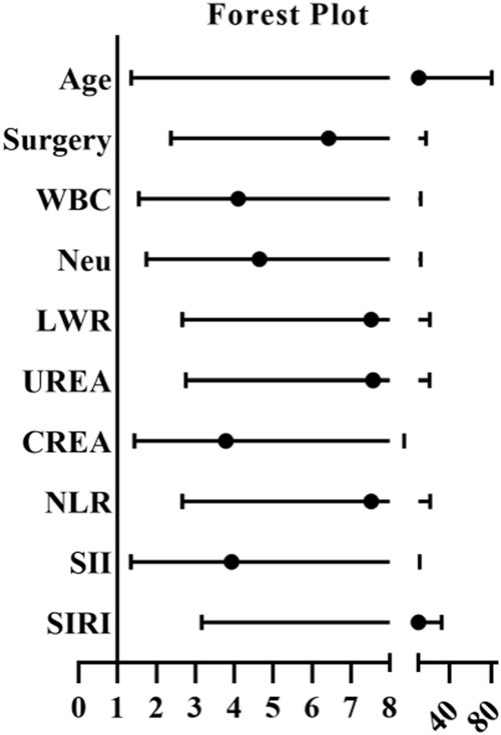
Surgery	6.431	2.371−17.478	<.001	
WBC	4.103	1.544−10.900	.008	
Neu	4.645	1.740−12.401	.003	
LWR	7.519	2.667−21.277	<.001	
UREA	7.571	2.759−20.782	<.001	
CREA	3.785	1.435−9.980	.008	
NLR	7.522	2.666−21.352	<.001	
SII	3.927	1.345−11.464	.014	
SIRI	10.111	3.163−32.306	<.001	

Abbreviations: CI, confidence interval; CREA, creatinine; HR, hazard ratio; LW = Lym/WBC; Neu, neutrophil; NLR = Neu/Lym; SII = PLT × Neu/Lym; SIRI = Neu × Mo/Lym; UREA, urea nitrogen; WBC, white blood cell.

**Table 4:** Multivariable logistic regression of in‐hospital mortality for patients with IE

Variables (SIRI)	HR	95% CI	*p *Value
Model 1	10.111	3.165−32.306	<.001
Model 2	8.966	2.753−29.204	<.001
Model 3	5.053	1.426−17.905	.012

*Note*: Model 1: we adjusted none; Model 2: we adjusted age and gender; Model 3: we adjusted age, gender, surgery.

Abbreviations: CI, confidence interval; CREA, creatinine; HR, hazard ratio; LWR = Lym/WBC; Neu, neutrophil; NLR = Neu/Lym; SII = PLT × Neu/Lym; UREA; urea nitrogen; WBC, white blood cells.

## 4 DISCUSSION

A total of 147 IE patients were included in this retrospective study. This study is the first to investigate the predictive value of SIRI level on the risk of in‐hospital death in IE patients. Our results showed that higher SIRI level was independently associated with the risk of in‐hospital death in IE patients, that is, SIRI was an independent predictor of the risk of in‐hospital death in IE patients.

Currently, the widely recognized prognostic factors of IE are mainly divided into four categories, including patient characteristics, cardiac or extracardial complications, pathogenic microorganisms, and echocardiographic findings.^8^ IE is a cardiovascular disease with high morbidity and mortality, which seriously threatens the life and health of patients. The mortality of IE within 1 year after diagnosis was close to 30%, and there was little improvement in the past 20 years,^9^, ^10^ and the mortality was as high as 8% 30 days after surgery.^11^ Therefore, early identification of IE risk factors and establishment of prognostic prediction model are of great significance.

WBC and its subsets are traditional inflammatory indicators and have been widely used in clinical. In recent years, new inflammatory markers, such as NLR, MLR, SII, SIRI, and so on have attracted the attention of many researchers because these markers are also easy to obtain and have more clinical value. SIRI is a new index derived by multiplying the Neu by the Mo and dividing it by the Lym, integrating three different inflammatory immune response mechanisms of Neu, Mo, and Lym. Existing research has shown that SIRI is associated with the progression and prognosis of a variety of diseases. Neu are the largest number of granulocytes in WBC, accounting for about 70% of the total number of WBC, mainly involved in the immune inflammatory response of the body. Mo are the largest WBC with a powerful phagocytosis function. In the process of inflammation, Mo differentiate into macrophages or dendritic cells after being regulated by local growth factors, proinflammatory, cytokines, and microbial products, which can effectively control and eliminate viruses, bacteria, and fungi.^12^ Compared with Mo, Lym are the smallest cells in WBC. Lym mainly include T lymphocytes and B lymphocytes, which participate in cellular immunity and humoral immunity, respectively. Lym can secrete specific antibodies or produce cytotoxicity to participate in immune inflammatory reactions.^13^ SIRI combines all three to represent the balance between inflammatory activators and inflammatory regulators. The higher the ratio, the greater the imbalance, indicating the more severe of the inflammatory response, the stronger the immunosuppression. SIRI was also shown to be associated with CRP levels, suggesting that SIRI is indeed an indicator of inflammation levels in the body.^14^ Li et al.^15^ believed that SIRI, as an indicator representing various inflammatory responses in the body, was an independent risk factor affecting the prognosis of non‐small cell lung cancer. Hua et al.^16^ pointed out that SIRI could be used to predict the effective prognostic factors of postmenopausal breast cancer undergoing simultaneous surgical treatment, and the overall survival of patients with higher SIRI value would be worse. Xu et al.^6^ conducted a retrospective study of 351 patients with liver cancer in the Cancer Research Center of Fudan University and found that SIRI was an independent risk factor affecting the prognosis of liver cancer and could be used as an effective indicator to predict the prognosis of liver cancer. Similarly, we also found that there is a positive relation between high SIRI and in‐hospital mortality.

In this study, univariate analysis showed that age, surgery, WBC, Neu, LWR, UREA, CREA, NLR, SII, and SIRI were associated with an increased risk of death in IE patients. When the above factors were included in multivariate analysis, SIRI was still significant, In addition, the performance of SIRI was well. Thus, this study confirmed that the SIRI is a risk factor of in‐hospital death for patients with IE.

Our research also has some limitations and shortcomings. First of all, this study was a single‐center retrospective study with a relatively small sample size, which may lead to selection bias and information bias. Second, since this study is a retrospective and observational study, we can only establish a correlation from it, rather than a causal relationship. In addition, due to the limitation of sample size, we only adjusted some of the more important factors related to the prognosis of IE patients, and some confounding factors may not be completely excluded. Finally, lacking data on several traditional markers of inflammation. In summary, the correlation between SIRI and the prognosis of IE patients needs further in‐depth research, especially multicenter, prospective, large‐sample clinical researches are more convincing.

In conclusion, the results of this study suggest that higher baseline SIRI level is an independently associated with the risk of in‐hospital death in IE patients and can be an independent predictor of poor outcome in IE patients. Using SIRI as a risk stratification indicator to identify the high‐risk IE patients early and carry out timely and effective clinical intervention, which could improve the quality of life and prognosis of IE patients.

## CONFLICTS OF INTEREST

The authors declare no conflicts of interest.

## Data Availability

The data sets used and/or analyzed during the current study areavailable from the corresponding author on reasonable request.
